# DId I Do the Right Thing? Moral and Ethical Dilemmas in Oncology Family Caregiving

**DOI:** 10.1093/geroni/igaf122.3568

**Published:** 2025-12-31

**Authors:** Jacquelyn Benson, Christi Lero, J Peter Siriprakorn, Anaika Bedi, Nashua Haque

**Affiliations:** Washington University in St. Louis, St. Louis, Missouri, United States; Washington University in St. Louis, St. Louis, Missouri, United States; Washington University in St. Louis, Saint Louis, Missouri, United States; Washington University in St. Louis, St. Louis, Missouri, United States; Georgetown University School of Medicine, Washington D.C., District of Columbia, United States

## Abstract

Family caregivers are central to cancer care, yet many difficulties extend beyond logistics of caregiving to dilemmas marked by moral uncertainty and ethical tension. Such dilemmas require value trade-offs rather than straightforward problem solving. U.S. research richly documents caregiver challenges but seldom characterizes moral/ethical dilemmas as caregivers themselves experience them. We report preliminary descriptive findings toward a caregiver-informed typology of moral and ethical dilemmas in cancer caregiving. Within an ongoing qualitative study, we conducted inductive thematic analysis of the initial interviews (20% of total sample). Two coders independently reviewed transcripts, wrote reflexive memos, iteratively refined a codebook, and resolved differences by consensus. This analysis was restricted to segments coded as moral or ethical dilemmas; purely practical challenges without explicit value/principle conflict were noted but not analyzed here. Four moral themes emerged: (1) truth-hope stewardship (balancing honesty with preserving hope); (2) rescue vs acceptance near end of life (e.g., whether to call 911 or escalate care); (3) good-caregiver identity vs. self-protection (managing others’ judgements while guarding emotional limits); and (4) goal-concordance uncertainty when preferences were unknown. Two ethical tensions were prominent: surrogate autonomy vs. best interest and decisional capacity/understanding at treatment turning points. Hybrid cases (moral + ethical) were common, and moral residue (“what ifs”) frequently followed decisions. Preliminary findings indicate caregiver routinely face moral/ethical dilemmas distinct from logistical barriers. A caregiver-informed typology can guide targeted supports (e.g., disclosure coaching, crisis planning, advance-care-planning prompts, post-decision debriefing) and sets up subsequent analyses of how caregivers navigate dilemmas and what impedes coping.

